# Storage related haematological and biochemical changes in *Plasmodium falciparum* infected and sickle cell trait donor blood

**DOI:** 10.1186/s12878-018-0128-x

**Published:** 2018-11-06

**Authors:** Enoch Aninagyei, Emmanuel Tetteh Doku, Patrick Adu, Alexander Egyir-Yawson, Desmond Omane Acheampong

**Affiliations:** 1 Department of Biomedical Sciences, School of Allied Health Sciences, University of Cape Coast, Cape Coast, Ghana; 2 School of Public Health, University of Ghana, Legon, Accra Ghana; 3 Department of Medical Laboratory Technology, University of Cape Coast, Cape Coast, Ghana

**Keywords:** Sickle cell trait donor, Asymptomatic malaria donor, % haemolysis, Storage lesions, Plasma haemoglobin, Biochemical changes, Blood transfusion

## Abstract

**Background:**

In sub-Saharan Africa where sickle cell trait (SCT) and malaria is prevalent, significant proportions of blood donors may be affected by one or more of these abnormalities. The haemato-biochemical properties of SCT and asymptomatic malaria in donor blood have not been evaluated. This study evaluated the haemato-biochemical impact of SCT and asymptomatic malaria infections in citrate-phosphate-dextrose-adenine (CPDA-1) stored donor blood units.

**Methods:**

Fifty-milliliters of sterile CPDA-1 anti-coagulated blood were drained into the sample pouch attached to the main blood bag. Ten units each of sickle cell/malaria negative, sickle cell and malaria positive blood were analyzed. Baseline and weekly haematological profiling and week 1, 3 and 5 concentrations of plasma haemoglobin, % haemolysis, sodium, potassium and chloride and lactate dehydrogenase (LDH) were assayed. Differences between baseline and weekly data were determined using one-way analysis of variance (ANOVA) and Kruskal-Wallis test, whereas differences between baseline parameters and week 1–3 data pairs were determined using paired t-test. *P*-value < 0.05 was considered statistically significant.

**Results:**

Storage of SCT and malaria infected blood affected all haematological cell lines. In the SCT donors, red blood cells (RBC) (4.75 × 10^12^/L ± 1.43^baseline^ to 3.49 × 10^12^/L ± 1.09^week-5^), haemoglobin (14.45 g/dl ± 1.63^baseline^ to 11.43 g/dl ± 1.69^week-5^) and haematocrit (39.96% ± 3.18^baseline^ to 33.22% ± 4.12^week-5^) were reduced. In the asymptomatic malaria group, reductions were observed in RBC (5.00 × 10^12^/L ± 0.75^baseline^ to 3.72 × 10^12^/L ± 0.71^week-5^), haemoglobin (14.73 g/dl ± 1.67^baseline^ to 11.53 g/dl ± 1.62^week-5^), haematocrit (42.72% ± 5.16^baseline^ to 33.38% ± 5.80^week-5^), mean cell haemoglobin concentration (35.48 g/dl ± 1.84^baseline^ to 35.01 g/dl ± 0.64^week-5^) and red cell distribution width coefficient of variation (14.81% ± 1.54^baseline^ to 16.26% ± 1.37^week-5^). Biochemically, whereas plasma LDH levels significantly increased in asymptomatic malaria blood donors (319% increase at week 5 compared to baseline), SCT blood donors had the most significant increase in plasma potassium levels at week 5 (382% increase). Sodium ions significantly reduced in SCT/malaria negative and sickle cell trait blood at an average rate of 0.21 mmol/L per day. Moreover, elevations in lymphocytes-to-eosinophils and lymphocytes-to-neutrophils ratios were associated with SCT and malaria positive blood whilst elevation lymphocytes-to-basophils ratio was exclusive to malaria positive blood.

**Conclusion:**

Severe storage lesions were significant in SCT or malaria positive donor blood units. Proper clinical evaluation must be done in prospective blood donors to ensure deferral of such donors.

**Electronic supplementary material:**

The online version of this article (10.1186/s12878-018-0128-x) contains supplementary material, which is available to authorized users.

## Background

There are two major outcomes of *Plasmodium* infections, thus symptomatic and asymptomatic infections. Symptomatic infections occur in patients with compromised anti-disease immunity [1] and asymptomatic infections occur in individuals with competent malaria immunity [2]. Despite being asymptomatic, parasites may be present in red blood cells, but at low density and can persist for many months [3]. Prevalence of asymptomatic *Plasmodium* infections differs from one geographical region to another. In Brazil, Solomon Islands and Cambodia, asymptomatic malaria has been found to be 37.5, 82.2 and 92% respectively [4–6]. In Africa, the prevalence of asymptomatic malaria in blood donors in Ghana has been found to be 10.0% [7]. In Cameroon, Senegal, Benin and Nigeria, asymptomatic malaria parasitaemia in blood donors have been found to be 27.54% [8], 65.3% [9], > 30% [10] and 40% [11] respectively.

Sickle cell haemoglobin result when valine substitutes for glutamic acid at position 6 of the β-globin chain. This mutation consequently changes the physico-chemical properties of the haemoglobin molecule [12]. A person who inherits haemoglobin S from one parent and haemoglobin A (normal gene) from the other has a sickle cell trait (SCT) or is said to be a carrier of sickle cell disease [13]. The prevalence of sickle cell trait (SCT) is highest in Africans and people of African descent. In Africa, Nigeria probably has the highest number of people with SCT as close to 30% of the population carry haemoglobin S gene [14]. Liberia, Ghana and Uganda have 10–15% of their population being carriers of haemoglobin S gene [13, 15, 16]. Cameroon and Gabon have haemoglobin S gene prevalence of 19 and 22%, respectively [17, 18]. SCT individuals are clinically healthy to donate blood [19]. Populations with high prevalence of sickle cell trait and asymptomatic *Plasmodium falciparum* infections can result in high number of healthy blood donors with sickle cell trait and/or asymptomatic *Plasmodium* infections. In Kenya and Nigeria, proportion of their blood donor population who were sickle cell carriers were 3.9 and 27.1% respectively [20, 21]. In Ghana, studies done in Brong Ahafo and Greater Accra regions reported prevalence of SCT in blood donors to be 12.5 and 11.3% respectively [12, 22].

Majority of the blood banks in SSA still practice whole blood banking [23, 24]. Although donated blood units are stored in anticoagulants supplemented with phosphate, dextrose, and/or adenine to assure long-term viability, storage lesions are inevitable when blood is stored for long periods [25]. RBC storage lesions include decreased RBC stability, alterations in various metabolites and the metabolic status of the cell, including decrease of intracellular adenosine triphosphate (ATP) and 2,3-biphosphoglycerate (2,3 BPG) [26–28]. These changes in RBC during storage have been known for years but the exact changes conferred on blood collected from SCT donors and those infected with *Plasmodium* parasites are unknown. The aim of this study therefore was to evaluate, for the first time, the haematological and biochemical variations in sickle cell trait blood and blood infected with *Plasmodium falciparum* stored in CPDA-1 anticoagulant up to 35 days.

## Methods

### Study design

This cross-sectional laboratory based experimental study was done in sterile blood units. Donors were selected according to Medical History Guide for Donor Selection [29].

### Donor selection, phlebotomy and specimen collection

The selections of the healthy blood donors were double-blinded as researchers and National Blood Service Ghana staff could not link the study specimens to any donor. Participant consent form was signed by all participated donors. Blood was collected from each of the donors following a modification of the technique described by Cheesbrough [25]. For the purpose of the study, 7 ml of the CDPA-1 anticoagulant was allowed into the sample pouch attached to the main blood bag, which was filled with the initial 50 ml of whole blood. The rest of the whole blood was directed into the main blood bag.

### Inclusion criteria

Donor blood included in the study were TTIs-negative blood with baseline biochemical parameters that fell within these ranges; haemoglobin 12.5–18.00 g/dl, plasma haemoglobin-0 g/dl, percentage haemolysis-0.0%, plasma sodium, potassium, chloride and LDH that fell within these respective ranges 135-145mmo/l, 3.0–5.2 mmol/l, 95-108 mmol/l and 100-250 U/L.

### Laboratory procedures

Post-phlebotomy laboratory procedures were done as follows: 5 ml of well mixed whole blood was aspirated into plain glass vacutainer tubes (All-Pro, Dusseldorf, Germany). Portion of the blood was used to screen for transfusion-transmitted infections (TTIs) using fourth generation enzyme immuno-assay (Abnova, Taiwan). The ELISA microtitre plate wells were pre-coated with hepatitis B surface monoclonal antibodies, gp36/gp41, hepatitis C antigens (core, NS3 and NS5) and *Treponema pallidum* antigens for qualitative detection of hepatitis B virus, HIV I&II, hepatitis C virus and *Treponema pallidum* respectively. Donor screening of sickle cells was done as described by Antwi-Baffour [13] whilst phenotyping was done in alkaline medium (pH = 8.6). Separation of haemolysate was done on cellulose acetate paper using 250 V voltage and 50 mA current for 30 min. The separated haemoglobin molecules were interpreted against pooled haemoglobin AFSC controls. Screening of malaria was done with PfHRP-2/Pf-LDH SD Bioline rapid diagnostic test kit (Gyeonggi-do, Republic of Korea). The kit detected *Plasmodium falciparum* specific HRP-2 proteins and lactate dehydrogenase enzymes. Five microliters of whole blood were dropped on the sample column of the rapid test kit. Four drops of assay diluent (PfHRP-2/Pf-LDH SD Bioline, Korea) were added to the buffer window of the kit. Results were read after 15 min. Baseline haematological parameters using 5-part differential Urit-5160 (Guangxi, China), plasma electrolytes (sodium, potassium and chloride) measured by FT-320 electrolyte analyzer (China), lactate dehydrogenase (measured by PKL-125 Italia fully automated chemistry analyzer using ELItech LDH kinetic reagent, France), plasma haemoglobin (Urit-5160, China) and percentage haemolysis calculated by using formulae used by Sawant et al. [30] was done on same day of blood collection (baseline). The donor whole blood study samples were stored in the blood bank refrigerator (Fiocchetti Scientific Refrigerator, Italy) for 5 weeks. Internal storage temperature was taken twice daily. Haematological parameters were determined weekly for 5 weeks whilst plasma haemoglobin, % haemolysis, plasma electrolytes and LDH were measured at the end of week 1, week 3 and week 5.

### Data processing and statistical analysis

Baseline and weekly data was entered into Microsoft Excel 2010. Statistical analysis was done by SPSS Version 24 (Chicago, IL, USA). Based on SCT status and/or asymptomatic malaria infection status, participants were grouped into three: SCT blood donors, asymptomatic malaria blood donors and no SCT/asymptomatic malaria blood donors for subsequent analysis. Differences between baseline and the 5 weeks haematological profile and biochemical data set were determined by one-way analysis of variance (ANOVA) and Kruskal-Wallis test statistical models. Differences between baseline haematological parameters and week 1 to 3 data pair were determined by paired student T-test. *P*-value of < 0.05 was considered statistically significant.

## Results

### Storage related changes in TWBC and WBC differential counts

The study recorded significant changes in total white blood cells (TWBC) and WBC differential in all the three donor groups (Table 1). There were consecutive reductions in TWBC and neutrophil proportion in all donor groups. Whereas the largest significant TWBC reduction occurred in the sickle cell/malaria negative group (62.3% reduction in week 5), the largest significant reduction in neutrophil% occurred in the SCT donors (53.9% reduction in week 5). However, there were successive increases in %lymphocytes, basophils and monocytes in all donor groups when weekly estimates were compared to baseline values. Whereas the highest increase in %lymphocytes occurred in the sickle cell/malaria negative donors (84.3% increase compared to baseline), the highest %monocyte increase occurred in the SCT donor group (75.6% increase compared to baseline). Additionally, although there was a consecutive reduction in %eosinophil count in the SCT group, the changes in %eosinophil count fluctuated in the sickle cell/malaria negative, and the asymptomatic malaria donor groups.

**Table 1:** Leukocytes and percentage differential storage changes in donor blood

TWBC and differentials	Baseline Mean	Week 1 Mean (%∆)	Week 2 Mean (%∆)	Week 3 Mean (%∆)	Week 4 Mean (%∆)	Week 5 Mean (%∆)	*P*-value
Sickle cell/malaria negative donor blood							
TWBCx10^9^/L	5.63	3.89(−30.9)	2.90(−48.4)	2.49(−55.7)	2.27(− 59.6)	2.09(−62.3)	0.001^*,a^
Neut %	66.18	60.09(− 9.2)	52.77(−20.2)	49.49(− 25.2)	44.21(− 33.2)	40.88(− 38.2)	0.054^b^
Lymp %	27.28	33.42(22.5)	39.83(46.0)	41.92(53.6)	46.94(72.0)	50.28(84.3)	0.092^b^
Eos %	3.03	2.73(−9.9)	2.42(−20.1)	3.32(9.5)	3.00(−0.9)	3.49(15.2)	0.845^a^
Mon %	3.27	3.52(7.6)	4.64(41.9)	4.94(51.1)	5.50(68.2)	4.96(51.6)	0.009^b^
Bas %	0.24	0.25(4.1)	0.35(45.8)	0.33(37.5)	0.35(45.8)	0.39(62.5)	0.033^b^
Sickle cell trait donor blood							
TWBCx10^9^/L	6.36	5.08 (−20.1)	4.42 (−30.5)	4.24 (−33.3)	3.74 (−41.2)	3.57 (−43.8)	0.001^*,b^
Neut %	56.26	50.91 (−9.5)	39.58(−29.6)	32.64 (−41.9)	31.99 (− 43.1)	25.90 (− 53.9)	0.001^*,b^
Lymp %	37.50	43.06 (14.8)	51.48 (37.3)	61.46 (63.9)	62.79 (67.4)	66.28 (76.7)	0.001^*,1^
Eos %	3.34	2.45 (−26.6)	2.50 (− 25.1)	1.56 (−53.3)	1.69 (−49.4)	2.77 (−17.0)	0.020^a^
Mon %	2.71	3.59 (32.4)	3.94 (45.4)	4.02 (48.3)	3.24 (19.5)	4.76 (75.6)	0.179^b^
Bas %	0.20	0.20 (0.0)	0.38 (90.0)	0.32 (60.0)	0.30 (50.0)	0.29 (45.0)	0.069^b^
Asymptomatic malaria donor blood							
TWBCx10^9^/L	6.08	4.49 (−26.1)	4.13 (−32.1)	3.77 (−37.9)	3.54 (−41.7)	3.21 (−47.2)	0.001^a^
Neut %	72.09	60.89 (−15.5)	47.78 (−33.7)	39.79 (−44.8)	42.91 (−40.4)	36.07 (− 49.9)	0.001^*,b^
Lymp %	31.67	32.53 (2.7)	45.65 (44.1)	52.53 (65.8)	49.52 (56.3)	55.63 (75.6)	0.001^*,b^
Eos %	2.38	2.50 (5.0)	2.01 (−15.5)	2.26 (−5.0)	2.39 (0.4)	2.69 (13.0)	0.265^a^
Mon %	3.56	3.72 (4.5)	4.68 (31.4)	5.08 (42.6)	4.57 (28.4)	5.29 (48.6)	0.095^b^
Bas %	0.30	0.36 (20.0)	0.33 (10.0)	0.34 (13.3)	0.31 (3.3)	0.32 (6.6)	0.804^a^

Abbreviations: *TWBC* = total white blood cells, *Neut* = Neutrophils, *Lymp* = Lymphocytes, *Eos* = Eosinophils, *Mon* = Monocytes, *Bas* = Basophils, *%* = Percent, *L* = Liter, *ANOVA* = Analysis of variance, *SD* = Standard deviation

^*^p values less than 0.001, ^a^*p*-value determined by Kruskal-Wallis H test, ^b^*p*-value determined by one-way ANOVA

### Storage related changes in RBC and RBC indices

This study also found significant changes in RBC count and RBC indices in all the three donor groups (Table 2). In all donor groups, there were consecutive reductions of RBC count, haemoglobin level, and HCT in all successive weekly estimations compared to baseline values. Whereas the most significant reduction in RBC count occurred in the SCT blood donor group, (26.5% decrease compared to baseline), the highest reductions in haemoglobin and HCT occurred in the sickle cell/malaria negative blood donor group [26.5% (Hb), and 25.2% (HCT) reductions compared to baseline]. Also, RDW-CV% values consecutively increased in all donor groups with respect to successive weekly measurement. However, whereas weekly MCV and MCH values consistently decreased in asymptomatic malaria donor group, these values fluctuated in SCT or sickle cell/malaria negative donor groups.

**Table 2:** RBC and RBC indices storage changes in donor blood

RBC and RBC indices	Baseline Mean	Week 1 Mean (%∆)	Week 2 Mean (%∆)	Week 3 Mean (%∆)	Week 4 Mean (%∆)	Week 5 Mean (%∆)	*P*-values
Sickle cell and malaria negative donor blood							
RBC (×10^12^/L)	4.72	4.27 (−9.5)	4.08 (− 13.5)	4.03 (− 14.6)	3.98(−15.6)	4.04 (− 14.4)	0.147^a^
Hb (g/dl)	13.17	11.35 (−13.8)	10.49 (− 20.3)	9.97 (− 24.3)	10.06 (−23.6)	9.74 (−26.0)	0.022^a^
HCT (%)	37.54	33.52 (−10.7)	28.83 (− 23.2)	28.52 (−24.0)	28.48(− 24.1)	28.06(− 25.2)	0.013^a^
MCV (fL)	75.88	76.99 (1.5)	75.34 (−0.7)	74.37 (−1.9)	74.67 (−1.6)	75.22 (− 0.9)	0.952^a^
MCH (pg)	26.70	26.23 (−1.7)	27.61 (3.4)	25.85 (−3.1)	26.39 (−1.1)	26.14 (−2.0)	0.924^a^
MCHC (g/dl)	32.47	31.39 (−3.3)	33.58 (3.4)	31.90 (−1.7)	32.45 (−0.6)	31.92 (−1.6)	0.932^a^
RDW_CV (%)	14.14	14.14 (0.0)	14.20 (0.4)	15.02 (6.2)	14.63 (3.4)	14.76 (4.3)	0.486^b^
RDW_SD (fL)	32.38	32.38 (0.0)	33.55 (3.6)	31.76 (−1.9)	32.14 (−0.7)	30.17 (−6.8)	0.756^b^
Sickle cell trait donor blood							
RBC (×10^12^/L)	4.75	4.06 (−14.5)	3.58 (−24.6)	3.57 (−24.8)	3.56 (−25.0)	3.49 (−26.5)	0.016^a^
Hb (g/dl)	14.45	12.84 (−11.1)	12.47 (−13.7)	11.47 (−20.6)	11.57 (− 19.9)	11.43 (−20.8)	0.002^a^
HCT (%)	39.96	36.75 (−8.0)	33.34 (−16.5)	33.29 (−16.7)	32.55 (− 18.5)	33.22 (− 16.8)	0.005^a^
MCV (fL)	82.54	86.71 (5.0)	82.02 (−0.6)	81.27 (−1.5)	82.03 (− 0.61)	82.95 (0.4)	0.573^a^
MCH (pg)	30.36	30.29 (−0.2)	30.39 (0.3)	28.54 (−5.9)	29.12 (−4.0)	28.43 (−6.3)	0.424^a^
MCHC (g/dl)	36.75	34.99 (−4.7)	37.08 (0.9)	34.96 (− 4.8)	35.50 (−3.4)	34.30 (−6.6)	0.001^a^
RDW_CV (%)	14.75	14.75 (0.0)	14.91 (1.0)	15.30 (3.7)	15.03 (1.8)	23.68 (60.5)	0.518^b^
RDW_SD (fL)	35.59	35.59 (0.0)	36.54 (2.6)	33.80 (5.0)	34.06 (4.2)	31.83 (10.5)	0.082^b^
Asymptomatic malaria donor blood							
RBC (×10^12^/L)	5.00	4.48 (−10.4)	4.02 (−19.6)	4.00 (−20.0)	3.96 (−20.8)	3.72 (−25.6)	0.001^*,a^
Hb (g/dl)	14.73	13.06 (−11.3)	12.12 (−17.7)	11.82 (− 19.7)	11.98 (− 18.6)	11.53 (−21.7)	0.001^*,a^
HCT (%)	42.72	38.83 (−9.1)	34.04 (−20.3)	34.32 (− 19.6)	34.33 (−19.6)	33.38 (−21.8)	0.001^*,a^
MCV (fL)	89.15	87.66 (−1.6)	82.04 (−7.9)	81.81 (−8.2)	82.68 (−7.2)	83.31 (−6.5)	0.067^a^
MCH (pg)	30.85	29.42 (−4.6)	29.66 (−3.8)	28.13 (−8.8)	29.20 (−5.3)	29.15 (−5.5)	0.320^a^
MCHC (g/dl)	35.48	33.57 (−5.3)	36.22 (2.0)	34.45 (2.9)	35.38 (−0.2)	35.01 (−1.3)	0.002^b^
RDW_CV (%)	14.81	14.81 (0.0)	15.80 (6.6)	16.39 (10.6)	16.16 (9.1)	16.26 (9.7)	0.024^a^
RDW_SD (fL)	36.53	36.53 (0.0)	35.75 (−2.1)	33.82 (−7.4)	34.49 (−5.5)	34.49 (−5.5)	0.267^a^

Abbreviations: *RBC* = Red blood cells, *Hb* = Haemoglobin, *HCT* = Haematocrit, *MCV* = Mean cell volume, *MCH* = Mean cell haemoglobin, *MCHC* = Mean cell haemoglobin concentration, *RDW_CV* = Red cell distribution width coefficient of variation, *RDW_SD* = Red cell distribution width standard deviation, *L* = Litre, *fL* = Fentolitre, *pg* = picogram

^*^p values less than 0.001; ^a^*p*-value determined by one-way ANOVA; ^b^*p*-value determined by Kruskal-Wallis H test

### Storage related changes in platelets and platelet indices

Changes in platelet count and related platelet indices were also observed in the donor population (Table 3). Consecutively, weekly platelet count estimates successively decreased in all blood donor groups; the highest significant reduction occurring in asymptomatic malaria blood donor group (49.9% decrease compared to baseline). Also, whereas weekly MPV and P_LCR successively increased in sickle cell/malaria negative and asymptomatic malaria donor group, the levels of these measurements fluctuated in the SCT blood donor group.

**Table 3:** Platelet and platelet indices storages changes in donor blood

	Baseline Mean	Week 1 Mean (%∆)	Week 2 Mean (%∆)	Week 3 Mean (%∆)	Week 4 Mean (%∆)	Week 5 Mean (%∆)	F	*P*-value
Sickle cell and malaria negative donor blood								
Plt (×10^9^/L)	224.10	199.55(10.9)	171.10(23.6)	162.60(27.4)	167.40(25.3)	139.30(37.8)	4.09	0.003
MPV (fL)	8.45	9.05(7.1)	9.93(17.5)	9.71(14.9)	9.17(8.5)	10.10(19.5)	8.18	0.001^*^
PDW (fL)	12.74	12.41(2.5)	13.93(9.3)	13.00(2.0)	11.98(5.9)	13.63(6.9)	1.00	0.425
PCT (%)	0.18	0.16(11.1)	0.17(5.5)	0.15(16.6)	0.15(16.6)	0.13(27.7)	1.09	0.374
P_LCR (%)	18.15	20.11(10.7)	21.86(20.4)	22.15(22.0)	19.21(5.8)	24.18(33.2)	1.24	0.304
Sickle cell trait donor blood								
Plt (×10^9^/L)	225.90	214.10(5.2)	185.20(18.0)	189.40(16.1)	207.80(8.0)	167.61(25.8)	1.25	0.301
MPV (fL)	8.69	8.53(1.8)	9.83(13.1)	9.78(12.5)	9.83(13.1)	10.45(20.2)	3.85	0.005
PDW (fL)	12.31	12.03(2.2)	13.89(12.8)	13.75(11.6)	13.72(11.4)	15.54(26.2)	1.55	0.189
PCT (%)	0.19	0.19(0.0)	0.18(5.2)	0.18(5.2)	0.20(5.2)	0.18(5.2)	0.31	0.903
P_LCR (%)	16.62	16.19(2.5)	20.02(20.4)	21.65(30.2)	20.35(22.4)	24.28(46.0)	2.09	0.080
Asymptomatic malaria donor blood								
Plt (×10^9^/L)	191.90	141.30(26.3)	132.70(30.8)	118.40(38.3)	106.80(44.3)	96.10(49.9)	7.47	0.001^*^
MPV (fL)	8.59	8.59(0.0)	9.87(14.9)	9.93(15.6)	9.10(5.9)	10.19(18.6)	2.52	0.040
PDW (fL)	13.90	13.90(0.0)	14.28(2.7)	14.75(6.1)	12.25(11.8)	15.98(14.9)	1.49	0.208
PCT (%)	0.15	0.15(0.0)	0.16(6.6)	0.15(0.0)	0.16(6.6)	0.14(6.6)	0.41	0.835
P_LCR (%)	19.48	19.48((0.0)	22.19(13.9)	22.17(13.8)	21.40(9.8)	26.89(38.0)	1.33	0.265

Abbreviations: *Plt* = Platelets, *PMV* = Mean platelet volume, *PDW*=Platelet distribution width, *PCT* = Plateletcrit, *P_LCR* = Platelet large cell ratio

^*^p values less than 0.001

### Storage related changes in leukocyte ratios

There was progressive increase in lymphocyte-to-eosinophils ratio (LER) in the three blood donor groups but the differences in the SCT/malaria negative group were not significant (%∆ 4.0–20.7%, F = 2.363, *p* = 0.052) whilst significant differences were seen in the SCT (%∆ 55.9–178%, F = 5.16, *p* = < 0.05) and the malaria positive donor group (%∆ 35.5–514.7%, F = 4.46, *p* = < 0.05). In addition to LER, significant increase in lymphocyte-to-basophils ratio (LBR) was observed in malaria positive donor group (%∆ 25.3–151.9%, F = 5.11, *p* = < 0.05) but not in SCT group (F = 0.73, *p* = 0.602) and SCT/malaria negative group (F = 0.34, *p* = 0.882). There were gradual increases in lymphocytes-to-monocytes ratio (LMR) in both the SCT and the malaria positive group but the differences between the values were not significant. Lymphocyte-to-neutrophil ratio (LNR) increased in the three groups but significant increases were seen in SCT (29.1–308.8%, F = 4.53, *p* = 0.001) and malaria positive blood (75–437.5%, F = 10.9, *p* < 0.05) (Table 4).

**Table 4:** Storage related changes in leucocyte ratios

Leukocyte ratio	Baseline Mean	Week 1 Mean(%∆)	Week 2 Mean(%∆)	Week 3 Mean(%∆)	Week 4 Mean(%∆)	Week 5 Mean(%∆)	F	*P*-value
Sickle cell and malaria negative donor blood								
LER	19.8	20.6(4.0)	20.3(2.5)	21.2(7.1)	22.5(13.6)	23.9(20.7)	2.36	0.052
LMR	9.7	10.0(3.1)	9.0(7.2)	8.7(10.3)	10.2(5.2)	10.0(3.1)	0.42	0.828
LBR	184	205(11.4)	128(30.4)	130(29.3)	150(18.5)	149(19.0)	0.34	0.882
LNR	0.73	0.83(13.7)	1.12(53.4)	1.17(60.3)	1.33(82.2)	1.88(157.5)	1.44	0.222
Sickle cell trait donor blood								
LER	11.8	18.4(55.9)	26.8(127.1)	36.1(205.9)	38.5(226.3)	32.8(178.0)	5.16	0.001*
LMR	12.3	13.1(6.5)	14.2(15.4)	16.9(37.4)	22.4(82.1)	13.9(13.0)	1.95	0.100
LBR	198	177(10.6)	141(28.8)	169(14.6)	183(7.6)	169(14.6)	0.73	0.602
LNR	0.79	1.02(29.1)	1.42(79.8)	2.22(181.0)	2.83(258.2)	3.23(308.8)	4.53	0.001
Asymptomatic malaria donor blood								
LER	6.8	9.2(35.3)	19.3(183.8)	19.0(179.4)	23.0(238.2)	41.8(514.7)	4.46	0.001
LMR	5.9	8.7(47.5)	10.6(79.7)	11.3(91.5)	10.6(79.7)	10.8(83.1)	2.07	0.083
LBR	79	99(25.3)	127(60.8)	175(121.5)	181(129.1)	199(151.9)	5.11	0.001*
LNR	0.32	0.56(75.0)	1.07(234.4)	1.42(343.7)	1.27(296.9)	1.72(437.5)	10.9	0.001*

Abbreviations: *LER*-lymphocytes-to-eosinophils ratio, *LMR*-lymphocytes-to-monocytes ratio, *LBR*-lymphocytes-to-basophils ratio, *LPR*-lymphocyte-to-platelets ratio, *LNR*-lymphocytes-to-neutrophils ratio

*p-value less than 0.001

Moreover, when the weekly estimates in haematological parameters were compared to baseline using paired t-test analyses, most of the significant changes occurred in the SCT blood donors or the asymptomatic malaria positive blood donors (Additional file 1: Table S1).

### Effect of storage on haemolytic and biochemical parameters in donor blood

The storage lesions related to haemolytic and biochemical parameters presented in Table 5. The plasma haemoglobin and %haemolysis consecutively increased with the successive weekly estimations in all blood donor groups. Additionally, weekly LDH and plasma potassium levels consecutively increased in weekly estimates compared to baseline in all blood donor groups. Whereas the most significant increase in LDH occurred in asymptomatic malaria blood donor group (319% increase over baseline), the most significant increase in plasma potassium levels occurred in SCT blood donor group (382% increase over baseline levels). However, weekly plasma sodium and chloride levels successively decreased in all blood donor group; the highest significant reductions occurring in asymptomatic malaria blood donor group [10.0% (sodium) and 21.3% (chloride) decreases compared to baseline].

**Table 5:** Analysis of haemolytic and biochemical parameters in donor blood stored for baseline, week 1, week 3 and week 5

Haemolytic parameters	Baseline Mean	Week 1 Mean (%∆)	Week 3 Mean (%∆)	Week 5 Mean (%∆)	*P*-value
Sickle cell and malaria negative donor blood					
Plasma Hb (g/dl)	0.00	0.10	0.11(9.1)^c^	0.21(52.4)^c^	0.001^*,a^
% Haemolysis	0.00	0.52	0.75(30.7)^c^	1.44(63.9)^c^	0.001^*,a^
LDH (U/L)	199.40	319.20 (60.1)	428.00 (114.6)	522.70 (162.1)	0.001^*,a^
Potassium (mmol/L)	4.14	5.65 (56.5)	7.72 (86.5)	10.03(142.3)	0.001^*,b^
Sodium (mmol/L)	137.94	134.52 (−2.5)	130.00 (−5.7)	126.33 (−8.4)	0.001^*,a^
Chloride (mmol/L)	98.66	89.06 (−3.5)	84.98 (−13.9)	83.55 (−15.3)	0.001^*,b^
Sickle cell trait donor blood					
Plasma Hb (g/dl)	0.00	0.24	0.37(35.1)^c^	0.46(47.8)^c^	0.001^*,b^
% Haemolysis	0.00	1.16	2.08(44.2)^c^	2.62(55.7)^c^	0.001^*,a^
LDH (U/L)	207.10	329.20 (59.0)	491.60 (137.4)	610.40 (194.7)	0.001^*,a^
Potassium (mmol/L)	4.27	6.95 (62.8)	12.14 (184.3)	20.58 (382.0)	0.001^*,a^
Sodium (mmol/L)	139.51	136.74 (−2.0)	133.67 (−4.2)	130.35 (−6.5)	0.001^*,a^
Chloride (mmol/L)	99.63	90.77 (−8.9)	83.99 (− 15.7)	78.50 (−21.2)	0.001^*,b^
Asymptomatic malaria donor blood					
Plasma Hb (g/dl)	0.00	0.22	0.26(15.4)^c^	0.32(31.3)^c^	0.001^*,a^
% Haemolysis	0.00	1.00	1.46(31.5)^c^	1.90(47.3)^c^	0.001^*,a^
LDH (U/L)	192.30	418.70 (117.7)	596.60 (210.4)	806.70 (319.5)	0.001^*,b^
Potassium (mmol/L)	4.38	8.38 (91.3)	11.66 (166.2)	15.01 (242.7)	0.001^*,b^
Sodium (mmol/L)	140.72	135.17 (−3.9)	130.16 (−7.5)	125.60 (−10.0)	0.001^*,a^
Chloride (mmol/L)	100.62	91.00 (−9.6)	85.71 (−14.8)	79.21 (−21.3)	0.001^*,a^

Abbreviations: *LDH* = Lactate dehydrogenase, *SD* = Standard deviation, *Hb* = Haemoglobin

^*^p values less than 0.001, ^a^*p*-value determined by Kruskal-Wallis H test, ^b^*p*-value determined by one-way ANOVA, ^c^ %Δ calculated with respect to week 1 mean values

### Comparison of haemolytic and biochemical parameters across blood donor groups

The storage lesions in donor blood were quantitatively compared across the groups with respect to the weeks in storage (Table 6). Plasma haemoglobin and %haemolysis were significantly higher in SCT blood donors with respective weekly measurements compared to the other blood donor groups. However, respective weekly plasma LDH levels were significantly higher in asymptomatic malaria blood donor group compared to respective values in the other blood donor groups. Additionally, although baseline plasma potassium levels were comparable in all blood donor groups, weekly plasma potassium levels were respectively higher in asymptomatic malaria or SCT blood donor group compared to sickle cell and malaria negative blood donor group. Moreover, plasma sodium and chloride significantly decreased in the asymptomatic malaria group compared to the other groups.

**Table 6:** ANOVA analysis of inter-donor category biochemical and haemolytic parameters

Parameters	Sickle cell/malaria negative donors Mean ± SD	Sickle cell trait donors Mean ± SD	Asymptomatic malaria donors Mean ± SD	F	*P*-value
Baseline					
Plasma Hb	0.00	0.00	0.00		
% haemolysis	0.00	0.00	0.00		
LDH (U/L)	202.45 ± 20.51	202.90 ± 18.91	173.20 ± 63.74	1.76	0.188
Potassium (mmol/L)	4.15 ± 0.70	4.22 ± 0.71	4.01 ± 1.50	0.39	0.681
Sodium (mmol/L)	138.44 ± 2.40	139.52 ± 2.54	126.37 ± 44.53	3.16	0.057
Chloride (mmol/L)	98.78 ± 1.66	99.88 ± 1.78	90.37 ± 31.87	2.68	0.085
Week 1					
Plasma Hb	0.10 ± 0.00	0.25 ± 0.13	0.20 ± 0.09	23.6	0.001^*^
% haemolysis	0.51 ± 0.05	1.12 ± 0.27	1.01 ± 0.35	20.7	0.001^*^
LDH (U/L)	318.36 ± 29.30	346.00 ± 78.68	370.90 ± 137.89	14.6	0.001^*^
Potassium (mmol/L)	5.54 ± 0.41	7.02 ± 1.15	8.46 ± 0.75	33.6	0.001^*^
Sodium (mmol/L)	134.90 ± 3.06	136.95 ± 2.88	121.09 ± 42.80	1.16	0.326
Chloride (mmol/L)	89.60 ± 3.12	90.53 ± 4.19	81.74 ± 28.74	1.47	0.246
Week 3					
Plasma Hb	0.11 ± 0.03	0.39 ± 0.12	0.23 ± 0.11	22.2	0.001^*^
% haemolysis	0.76 ± 0.35	2.21 ± 0.62	1.37 ± 0.41	17.3	0.001^*^
LDH (U/L)	436.36 ± 46.31	491.10 ± 47.17	605.77 ± 61.98	33.0	0.001^*^
Potassium (mmol/L)	8.05 ± 1.39	12.02 ± 1.61	11.83 ± 1.47	40.3	0.001^*^
Sodium (mmol/L)	130.88 ± 4.23	133.45 ± 3.42	129.00 ± 3.51	3.74	0.035
Chloride (mmol/L)	85.61 ± 3.91	83.37 ± 3.44	85.37 ± 2.85	0.73	0.487
Week 5					
Plasma Hb	0.20 ± 0.04	0.48 ± 0.19	0.28 ± 0.14	8.84	0.001^*^
% haemolysis	1.46 ± 0.51	2.75 ± 1.00	1.78 ± 0.65	4.48	0.001^*^
LDH (U/L)	536.09 ± 58.71	631.90 ± 109.29	798.00 ± 64.38	13.0	0.001^*^
Potassium (mmol/L)	11.35 ± 4.46	19.48 ± 2.86	15.18 ± 1.98	8.25	0.001^*^
Sodium (mmol/L)	127.64 ± 6.00	129.62 ± 5.17	124.73 ± 4.79	9.70	0.001^*^
Chloride (mmol/L)	84.27 ± 4.29	77.81 ± 4.31	78.61 ± 3.44	1.22	0.309

Abbreviations: *LDH* = Lactate dehydrogenase, *SD* = Standard deviation, *Hb* = Haemoglobin

^*^p-values less than 0.001

## Discussion

Preservation and long term storage of red blood cells is essential for continuous supply of safe blood for clinical use [24]. Previous studies have found deleterious storage effect on blood cell morphology and functions [31–33]. This study found similar trend of storage lesion in whole blood stored up to 5 weeks but the changes were more pronounced in blood collected from sickle cell trait (SCT) donors or donors infected asymptomatically with malaria parasites. In the three groups, gradual reduction in total white cells and neutrophils and progressive increase in lymphocytes were observed; a phenomenon that was previously observed by Adias [24]. This observation probably can be due to cell loss and cytotoxic effect of histamine and cytokines released by neutrophils [34]. TWBC, neutrophils and eosinophils cells lost were more evident in the SCT or asymptomatic malaria positive stored blood than sickle cell and malaria negative blood. It was observed that lymphocytes to eosinophils, monocytes, basophils and neutrophils ratios were elevated in the three groups but in all cases significant elevations occurred in SCT and malaria positive donor blood. Insignificant elevations were observed in SCT/malaria negative blood. Elevation in LER, LMR, LBR and LNR values occurred as a result of progressive elevations in mean lymphocytes percentages and reduction in mean absolute eosinophils, monocytes, basophils and neutrophils. This predisposes blood recipients to bacterial invasion and increased proliferation of pathogenic bacterial [35, 36]. Fever is a common symptom of sepsis [37]. Donor blood with high LER, LMR, LBR and LNR could cause pathogen induced acute or delayed febrile reactions in recipients with low leukocyte count.

In sickle cell and malaria negative group, red blood cells were relatively stable during storage but in the SCT or asymptomatic malaria donor group, there were significant reduction in red blood cells, haemoglobin and haematocrit. These haematological changes corresponded with gradual increases in plasma haemoglobin due to increased red cell breakdown as well as gradual elevation of potassium ions and lactate dehydrogenase during storage. These observations could be as a result of haemoglobin S erythrocytes fragility subsequent to polymerization of haemoglobin S and reduced deformability and loss of red cell elasticity during storage due to low oxygen tension and low pH storage medium [38–40] on the one hand, as well as metabolically active intra-erythrocytic *Plasmodium* parasites could account for these haematological changes. Malaria parasites have been found to be viable in stored blood for at least the first 14 days [41]. When one malaria parasite per microliter of blood is found, that converts to about 500,000 red cells parasitized in a unit of blood [42]. These viable parasites are enough to cause significant haematological derangement in the blood of an infected healthy donor during storage. One of the goals of haemo-transfusion is to restore tissue oxygenation [43]. Stored blood from SCT and asymptomatic malaria donors may develop storage lesions over time due to polymerization of haemoglobin S in SCT donor blood, loss of deformability and increased osmotic fragility which could compromise their haemorheological properties and oxygen binding and delivery capacity [44, 45].

Storage of blood collected from SCT and asymptomatic malaria donors were significantly associated with red cell lysis and elevated plasma haemoglobin. On day of blood collection, donor plasma was free of haemoglobin as well as insignificant differences in potassium, sodium, LDH and chloride in all the donor groups. However at week 1, plasma haemoglobin was 2.4 times higher in SCT donor blood and 2.2 higher in asymptomatic malaria blood compared to sickle cell and malaria negative group. At week 3, plasma haemoglobin was 3.36 times and 2.36 times higher in SCT and asymptomatic malaria donor blood respectively, and at week 5, plasma haemoglobin was 2.2 times higher in SCT and 1.5 higher in asymptomatic malaria group. Excess plasma haemoglobin increased over time in the SCT and the asymptomatic malaria donor groups. At week 1, the percentage haemolysis in the SCT and asymptomatic malaria was more than the permissible level of 0.8%. Blood with % haemolysis of 0.8% is not recommended for clinical use [30]. Excess haemoglobin has been found to have negative influence on the intravascular nitric oxide (NO) metabolism after transfusion. Plasma haemoglobin has been found to be a potent scavenger of NO, the most important endogenous vasodilator. In view of this, transfusing blood with high concentration of plasma haemoglobin could decrease NO bioavailability, decreased organ perfusion, increased organ injury [46, 47] and increased mortality in patients with sepsis [48]. Patients with organ failure and patients with septic shock may worsen their condition when transfused with blood with high plasma haemoglobin content. Potassium increased in the SCT and asymptomatic malaria groups and continued till their respective levels were 4.8 and 3.4 times higher than the baseline at week 5, a factor far more than was observed in the sickle cell/malaria negative group. It is recommended to include malaria and sickle cells screening into donor screening protocols to prevent potassium and free haemoglobin overload. In addition, the clinical impact of transmitting malaria to the recipient, albeit asymptomatic in the donor, merits consideration with recommendations to prevent such transmissions [49].

## Conclusion

Storage of SCT and malaria infected blood affected all the cell lines. At week 1, total white blood cells, neutrophils, red cells, haemoglobin and haematocrit began to fall significantly in SCT and asymptomatic donor blood. Significant elevation plasma haemoglobin, increase in % haemolysis above the permissible level of 0.8% and potassium elevation above the upper reference range for Ghanaian adults (5.2 mmol/L) [50] were observed. Significant reduction in red blood cells coupled with significant elevations in plasma haemoglobin, intracellular potassium and lactate dehydrogenase can led to the conclusion that significant number of red cells haemolysis in the experimental set up. This assumption was confirmed by steady elevation of % haemolysis over the storage weeks.

### Limitations of the study

Malaria parasites were not quantified in the infected blood units. The analysis did not take into consideration the blood group and Rhesus phenotypes of the study units. The gender of the blood donors was unknown. Haemoglobin S was not quantified in the sickle cell trait donor blood.

## Additional file

Additional file 1: **Table S1.** Paired sample analysis in haematological parameters among the groups: baseline vs. week 1–3. (DOCX 20 kb)

## Abbreviations

2,3 BPG: 2,3-biphosphoglycerate
ANOVA: One-way analysis of variance
ATP: Adenosine triphosphate
Bas: Basophils
CPDA: Citrate Phosphate Dextrose Adenine
Eos: Eosinophils
fL: Fentoliter
GHS: Ghana Health Service
Hb: Haemoglobin
HCT: Haematocrit
LBR: Lymphocytes-to-basophils ratio
LER: Lymphocytes-to-eosinophils ratio
LMR: Lymphocytes-to-monocytes ratio
LNR: Lymphocytes-to-neutrophils ratio
Lymp: Lymphocytes
MCH: Mean cell haemoglobin
MCHC: Mean cell haemoglobin concentration
MCV: Mean cell volume
Mon: Monocytes
NBSG: National Blood Service Ghana
Neut: Neutrophils
P_LCR: Platelet large cell ratio
PCT: Plateletcrit
PDW: Platelet distribution width
pg: pictogram
Plt: Platelets
PMV: Mean platelet volume
RBC: Red blood cells
RDW_CV: Red cell distribution width coefficient of variation
RDW_SD: Red cell distribution width standard deviation
SCT: Sickle cell trait
SD: Standard deviation
SSA: sub-Saharan Africa
TTIs: Transfusion transmitted infections
TWBC: Total white blood cell
