# Supplementary material for: Storage related haematological and biochemical changes in Plasmodium falciparum infected and sickle cell trait donor blood
Source: BMC Hematol. 2018 Nov 6;18:30. doi: 10.1186/s12878-018-0128-x (PMC6220467; doi:10.1186/s12878-018-0128-x)
Supplement: Supplementary file 1 — Table S1. Paired sample analysis in haematological parameters among the groups: baseline vs. week 1–3. (DOCX 20 kb) [file 12878_2018_128_MOESM1_ESM.docx]

**Table S1: Paired sample analysis in haematological parameters among the groups: baseline vs. week 1-3**

|  |  | | | Blood donor study groups | | |  | | |
| --- | --- | --- | --- | --- | --- | --- | --- | --- | --- |
|  | **Sickle cell/malaria negative** | | | **Asymptomatic malaria** | | | **Sickle cell trait** | | |
| Profile | **Baseline vs.**  **week 1** | **Baseline vs.**  **week 2** | **Baseline vs.**  **week 3** | **Baseline vs.**  **week 1** | **Baseline vs.**  **week 2** | **Baseline vs.**  **week 3** | **Baseline vs.**  **week 1** | **Baseline vs.**  **week 2** | **Baseline vs.**  **week 3** |
| TWBC (x10^9^/L) | 0.001* | 0.001 | 0.001 | 0.03 | 0.01 | 0.01 | 0.001* | 0.001* | 0.001* |
| Neut % | 0.114 | 0.001* | 0.030 | 0.001 | 0.001* | 0.001* | 0.173 | 0.001 | 0.001 |
| Lymp % | 0.054 | 0.011 | 0.033 | 0.001 | 0.001* | 0.001* | 0.031 | 0.005 | 0.002 |
| Eos % | 0.231 | 0.581 | 0.610 | 0.346 | 0.306 | 0.886 | 0.020 | 0.049 | 0.023 |
| Mon % | 0.471 | 0.407 | 0.006 | 0.334 | 0.020 | 0.014 | 0.019 | 0.020 | 0.089 |
| Bas % | 0.343 | 0.157 | 0.015 | 0.089 | 0.592 | 0.463 | * | 0.034 | 0.038 |
| RBC (x10^12^/L) | 0.06 | 0.002 | 0.002 | 0.005 | 0.001 | 0.001 | 0.056 | 0.001 | 0.001 |
| Hb (g/dl) | 0.051 | 0.032 | 0.001* | 0.001* | 0.001* | 0.001* | 0.088 | 0.002 | 0.001* |
| HCT (%) | 0.059 | 0.001 | 0.001 | 0.001* | 0.001* | 0.001* | 0.101 | 0.003 | 0.001* |
| MCV (fL) | 0.650 | 0.738 | 0.279 | 0.596 | 0.005 | 0.012 | 0.071 | 0.777 | 0.475 |
| MCH (pg) | 0.577 | 0.177 | 0.185 | 0.026 | 0.031 | 0.024 | 0.936 | 0.971 | 0.053 |
| MCHC (g/dl) | 0.053 | 0.061 | 0.296 | 0.016 | 0.042 | 0.080 | 0.107 | 0.627 | 0.018 |
| RDW_CV (%) | ** | 0.910 | 0.003 | <0.005 | 0.850 | 0.080 | 0.006 | 0.093 | 0.948 |
| RDW_SD (fL) | ** | 0.032 | 0.167 | 0.005 | 0.215 | 0.042 | 0.003 | 0.765 | 0.017 |
| Plt (x10^9^/L) | 0.253 | 0.055 | 0.001* | 0.001* | 0.001* | 0.001* | 0.177 | 0.002 | 0.008 |
| MPV (fL) | 0.051 | 0.010 | 0.025 | ** | 0.030 | 0.015 | 0.531 | 0.914 | 0.034 |
| PDW (fL) | 0.082 | 0.164 | 0.902 | ** | 0.791 | 0.539 | 0.007 | 0.497 | 0.500 |
| PCT (%) | 0.144 | 0.005 | 0.004 | ** | 0.474 | 0.008 | 0.06 | 0.004 | 0.011 |
| P_LCR (%) | 0.076 | 0.081 | 0.071 | ** | 0.412 | 0.446 | 0.049 | 0.204 | 0.088 |

** The t-test was not computed because the standard error of the difference was 0, *p values less than 0.001. Abbreviations: TWBC=total white blood cells, Neut=Neutrophils, Lymp=Lymphocytes, Eos=Eosinophils, Mon=Monocytes, Bas=Basophils, %=Percent, L=Liter, ANOVA=Analysis of variance, SD=Standard deviation, RBC=Red blood cells, Hb=Haemoglobin, HCT=Haematocrit, MCV=Mean cell volume, MCH=Mean cell haemoglobin, MCHC=Mean cell haemoglobin concentration, RDW_CV=Red cell distribution width coefficient of variation, RDW_SD=Red cell distribution width standard deviation, L=Litre, fL=Fentolitre, pg=pictogram, Plt=Platelets, PMV=Mean platelet volume, PDW=Platelet distribution width, PCT=Plateletcrit, P_LCR=Platelet large cell ratio
